# The Anti-Apoptotic Activity of BAG3 Is Restricted by Caspases and the Proteasome

**DOI:** 10.1371/journal.pone.0005136

**Published:** 2009-04-08

**Authors:** Victoria M. Virador, Ben Davidson, Josephine Czechowicz, Alisha Mai, Jareer Kassis, Elise C. Kohn

**Affiliations:** Molecular Signaling Section, Medical Oncology Branch, Center for Cancer Research, National Cancer Institute, Bethesda, Maryland, United States of America; Emory University, United States of America

## Abstract

**Background:**

Caspase-mediated cleavage and proteasomal degradation of ubiquitinated proteins are two independent mechanisms for the regulation of protein stability and cellular function. We previously reported BAG3 overexpression protected ubiquitinated clients, such as AKT, from proteasomal degradation and conferred cytoprotection against heat shock. We hypothesized that the BAG3 protein is regulated by proteolysis.

**Methodology/Principal Findings:**

Staurosporine (STS) was used as a tool to test for caspase involvement in BAG3 degradation. MDA435 and HeLa human cancer cell lines exposed to STS underwent apoptosis with a concomitant time and dose-dependent loss of BAG3, suggesting the survival role of BAG3 was subject to STS regulation. zVAD-fmk or caspase 3 and 9 inhibitors provided a strong but incomplete protection of both cells and BAG3 protein. Two putative caspase cleavage sites were tested: KEVD (BAG3^E345A/D347A^) within the proline-rich center of BAG3 (PXXP) and the C-terminal LEAD site (BAG3^E516A/D518A^). PXXP deletion mutant and BAG3^E345A/D347A^, or BAG3^E516A/D518A^ respectively slowed or stalled STS-mediated BAG3 loss. BAG3, ubiquitinated under basal growth conditions, underwent augmented ubiquitination upon STS treatment, while there was no increase in ubiquitination of the BAG3^E516A/D518A^ caspase-resistant mutant. Caspase and proteasome inhibition resulted in partial and independent protection of BAG3 whereas inhibitors of both blocked BAG3 degradation. STS-induced apoptosis was increased when BAG3 was silenced, and retention of BAG3 was associated with cytoprotection.

**Conclusions/Significance:**

BAG3 is tightly controlled by selective degradation during STS exposure. Loss of BAG3 under STS injury required sequential caspase cleavage followed by polyubiquitination and proteasomal degradation. The need for dual regulation of BAG3 in apoptosis suggests a key role for BAG3 in cancer cell resistance to apoptosis.

## Introduction

Apoptosis following unrecoverable stress results from the activation of proteolytic pathways, which orchestrate the loss of survival proteins. Survival proteins can be degraded directly by activated caspases responding to intrinsic or extrinsic stimuli [1], [2] or targeted by the ubiquitin proteasome pathway [3]. Interruption of either or both proteolytic pathways can revert the apoptotic process and result in cytoprotection.

BAG3 (NM_004281) was reported initially as a protein-refolding cochaperone of the bcl2 binding protein BAG family [4], [5] and as upregulated in response to persistent stress of cellular calcium balance dysregulation [6]. It has been shown to diminish stress-induced apoptosis [5], [7]. BAG family of proteins contains a conserved BAG domain that binds the ATPase of heat shock protein (Hsp70) [4], [8], [9]. At least two members of the mammalian BAG family are also involved in cytoprotection, BAG1 [8] and BAG4 [10], [11]. This functional redundancy suggests selective targets for the different family members, allowing the family broad potential to protect against varied stresses in different cellular contexts.

We have demonstrated that BAG3, through its interaction with Hsp70, overcame geldanamycin-driven proteasomal protein degradation [7]. Overexpression of BAG3 prevented or reduced destruction of polyubiquitinated Hsp90/hsp70 client proteins such as cyclin D1, AKT, glycogen synthase kinase 3β, and ^p70^S6 kinase, and facilitated cell survival [7]. The protective effect of BAG3 was also observed when cells were exposed to heat shock. We also analyzed whether BAG3 provided cytoprotection under intrinsic apoptotic pathway stimulation by staurosporine (STS). By comparison with those other cellular stresses, limited protection was observed with BAG3 overexpression, leading to our current hypothesis that BAG3 is itself lost under selected apoptotic stimuli.

We now report that BAG3 falls victim to STS-induced apoptosis. Loss of BAG3 through RNA silencing augmented STS-mediated apoptosis, whereas, preventing BAG3 proteotoxicity was associated with cytoprotection. We demonstrate a requirement for sequential caspase cleavage followed by ubiquitination and proteasomal degradation under STS stress. Interruption of both pathways is required to restore BAG3 and overcome the apoptotic drive. The need for this dual and sequential regulation of BAG3 suggests a selective survival role of BAG3 in the cancer cells.

## Results

### STS treatment results in degradation of BAG proteins

**Figure 1:**
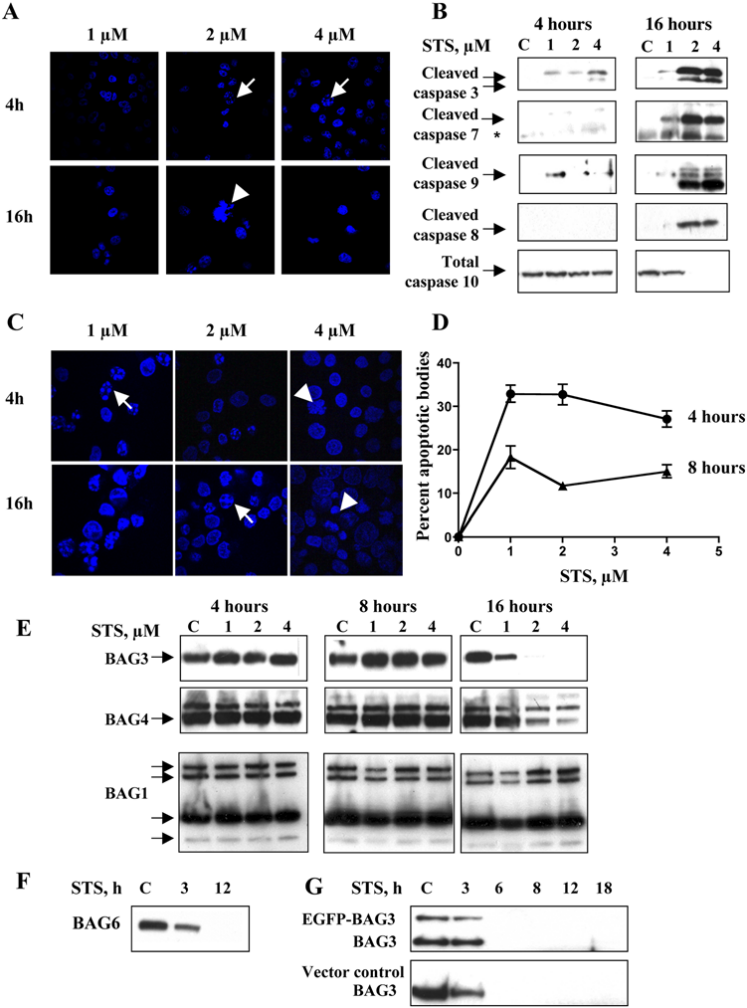
STS induced apoptosis and degradation of BAG3. A. STS induces cellular injury in a dose and time-dependent fashion. DAPI-stained STS-treated MDA435 cells show chromatin condensation consistent with apoptotic injury. Apoptotic bodies were observed with 2 and 4 µM STS at 4 hr (arrows) and there was a net loss of cells at 16 hrs. B. STS-mediated degradation of BAG3 occurs concomitant to activation of caspases 3, 7 and 9. Cleavage of caspases 3 and 9 were observed as early as 4 hrs into STS treatment. In comparison activation of caspases 8 and 10 was delayed, occurring after BAG protein loss was initiated. C, D. STS induces apoptosis in HeLa cells. Apoptotic bodies were observed in DAPI stained cells. Data points are the mean and SEM of five independent fields (n = 2). E, F. BAGs 3, 4, and 6 are lost progressively with STS treatment. Floating and adherent MDA435 cells were collected, lysed, and subjected to immunoblot. BAGs 3 and 4 (E), and 6 (F) were lost with STS exposures of 3–16 hrs. No reduction in the p50, p46, p34, or p29 BAG1 isoforms was observed (E). G. STS-mediated BAG3 degradation is neither cell line nor construct-specific. EGFP-BAG3 or EGFP-C1 empty vector were stably expressed in HeLa cells. Cells were exposed to 2 µM STS and both endogenous BAG3 and EGFP-BAG3 fusion proteins were lost over time, as early as 6 h.

STS caused dose- and time-dependent apoptosis in MDA435 human breast cancer cells (Figure 1A). Concomitant with nuclear condensation and cell death due to STS was progressive activation of caspases 3, 7, 8, 9, and 10 (Figure 1B). Caspases 3, 9 and 7 were cleaved earlier and at lower STS doses than caspases 8 or 10, confirming the expected predominant activation of the intrinsic apoptotic pathway. A similar effect was observed in HeLa cells. Apoptosis, demonstrated by the presence of apoptotic bodies, occurred earlier, at 4 and 8 hours (Figure 1C, D and Figure S2B, DMSO control). Cells lacked normal nuclear morphology at later time points, consistent with progressive injury (Fig 1C, arrow head). In BAG3 overexpressing HeLa and MDA435 cells, BAG3 colocalized with active mitochondria early in STS-mediated injury (Figure S1, arrows). Higher concentrations of, or longer exposure to, STS resulted in a generalized uptake of Mito-Tracker into the nucleus, indicating the lack of mitochondrial membrane integrity seen in apoptosis (Figure S1). The dose and time course of activation of caspases 3, 9 and 7 (Figure 1B) paralleled the progressive loss of BAG3 (Figure 1E). Family members BAGs 4, and 6 were similarly lost with STS treatment (Figure 1E, F) as were the commonly used ‘housekeeping proteins’ GAPDH and β-tubulin (Figure S5), while the four isotypes of BAG1, ^p50^BAG1L, ^p46^BAG1M, ^p34^BAG1, and ^p29^BAG1S, were unaffected, arguing against a global toxic effect of STS. Both endogenous and forced BAG3 were susceptible to this proteotoxicity. HeLa cells stably expressing EGFP-BAG3 displayed time-dependent loss of the fusion protein, as well as endogenous BAG3 (Figure 1G). The parallel between caspase activation and loss of BAGs 3, 4, and 6 suggested that intrinsic pathway caspases might be involved in BAG3 degradation.

### Silencing BAG3 sensitizes cells to STS-induced apoptosis

**Figure 2:**
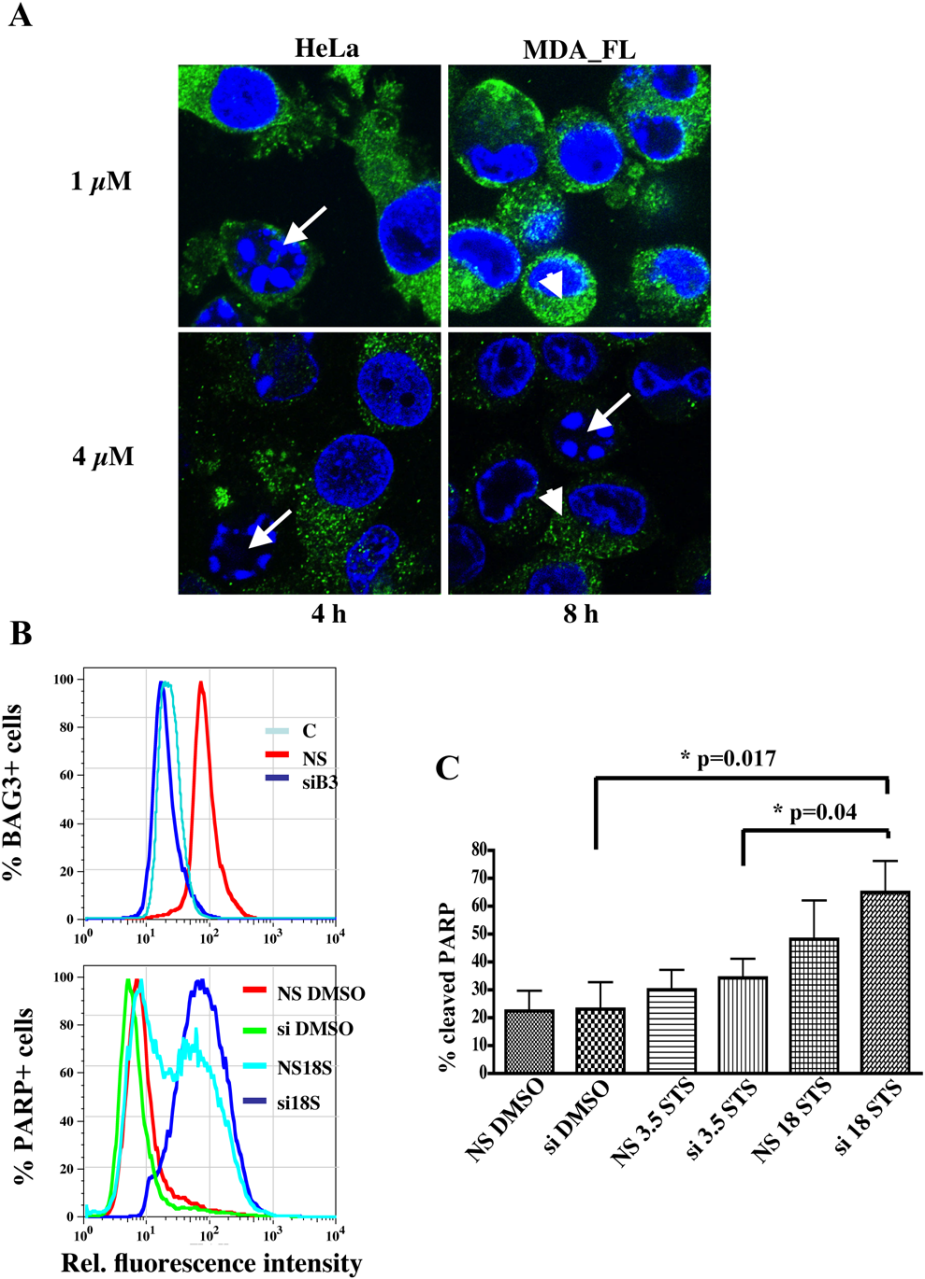
Loss or silencing of BAG3 sensitizes cells to apoptosis. A. Cells with intact nuclear morphology retain BAG3 signal. In HeLa with basal expression of BAG3, the appearance of apoptotic bodies (arrows) correlates with the loss of BAG3 green signal (arrowhead) in individual cells. In MDA_FL with forced expression of BAG3 the same phenomenon occurs although at higher STS dose and exposure time. B. PARP cleavage is augmented with BAG3 silencing and progressive STS dose. Adhered and floating MDA435 cells were stained with FITC-conjugated anti-cleaved PARP and anti BAG3 antibodies. Top graph shows BAG3 silencing by the leftward shift of the BAG3 positive population (dark blue line). Bottom graph shows the PARP+ population with BAG3 silencing and 18 h STS exposure (si 18S, dark blue line), compared with 18 h STS exposure but no silencing (NS 18STS, light blue line) along with controls (NS DMSO, si DMSO). C. Quantitation of percent apoptosis after 3.5 or 18 h of STS exposure from the cleaved PARP population (median FL-1) in response to BAG3 silencing combined with STS exposure (3.5 h and 18 h); data represent mean and SEM (n = 3).

Cells with greater expression of BAG3 were less likely to succumb to STS apoptosis. BAG3 signal was lost first in cells that had nuclear evidence of apoptosis (Figure 2A arrows) yet remained detectable in their morphologically normal neighbors (Figure 2A arrow head). This suggested that loss of BAG3 might be required for STS-mediated cell death. The green BAG3 cytoplasmic signal disappeared at higher STS dose or with longer treatment time in MDA435 cells with forced expression of BAG3 than in wild type HeLa cells with less endogenous BAG3. Direct fluorescence comparison between apoptototic and non-apoptotic cells is difficult due to their different cytosolic volume, thus we followed on this observation by investigating whether silencing BAG3 had an effect on apoptosis. Silencing BAG3 (Fig 2B, top panel and Figure S4) sensitized cells to STS-induced apoptosis, as demonstrated by progressive PARP cleavage under increasing STS exposure and silenced BAG3 (Figure 2B, bottom panel and quantitation in Fig 2C). We observed a consistent but transient induction of BAG3 around 4 hours into STS exposure (Figure S2), suggesting an attempt by the cells to resist injury. However, these levels, even with the brief induction, are not sufficient to overcome cellular commitment to apoptosis. These data indicate loss of BAG3 is necessary but may not be sufficient for STS induced apoptosis.

### BAG3 is a caspase substrate

**Figure 3:**
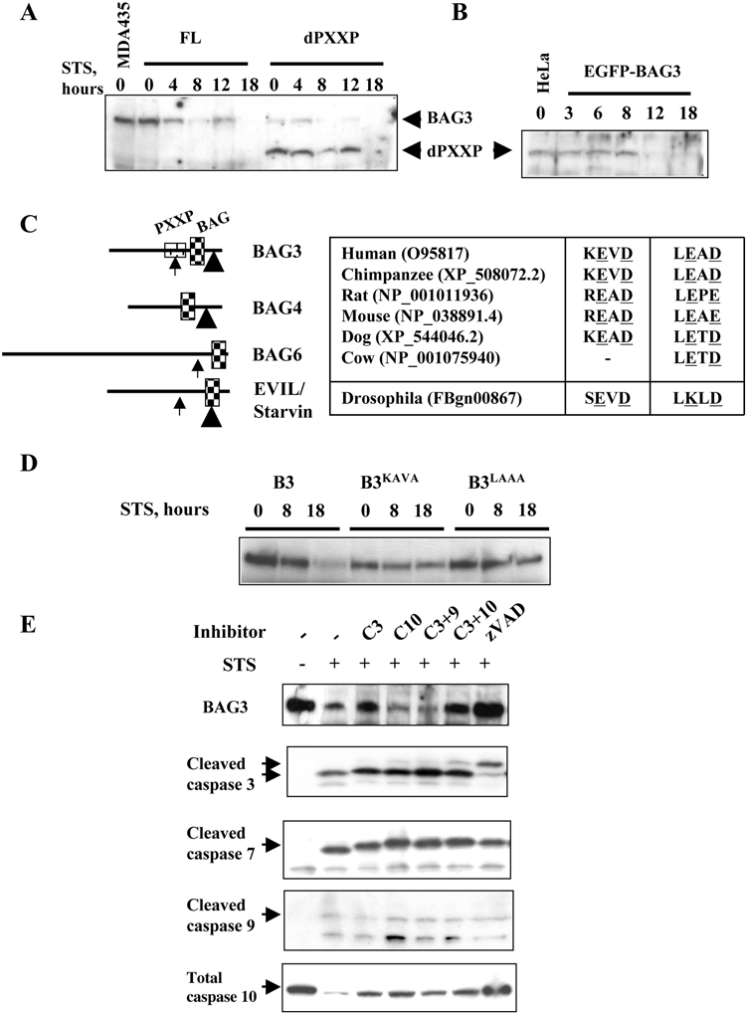
BAG3 is a direct caspase substrate. A, B. Deletion of the BAG3 PXXP domain delays loss of BAG3. Clonal FL-BAG3 and (d)PXXP-BAG3 MDA435 cells were treated with 2 µM STS as indicated. Cells were lysed and subjected to immunoanalysis with anti-BAG3. Delayed loss of dPXXP-BAG3, which contains putative caspase recognition sequence KEVD, is demonstrated compared with wild type protein. The delayed loss of dPXXP-BAG3 is confirmed in HeLa cells engineered to stably express EGFP-dPXXP-BAG3 by anti-EGFP (B). C. Location and alignment of putative caspase cleavage sites in BAGs 3, 4 and 6. KEVD (caspase 3 recognition site in the PXXP domain; arrow) and LEAD (caspase 9 recognition site; arrow head). Similar sites, represented to approximate scale, are found in BAGs 4 (^421^LELD) and 6 (^998^DEQD) [14], [37], but not in BAG1. These putative caspase cleavage sites are conserved in mammalian BAG proteins and in a Drosophila homolog (inset table). D. Mutation of either putative caspase cleavage site reduced loss of BAG3. Alanine mutagenesis of ^344^KEVD or ^515^LEAD was followed by stable transfection and expression. BAG3 was markedly decreased in B3 cells in response to 2 µM STS at the indicated times. BAG3^KAVA^ cells retained much of their BAG3 protein up to 18 hr STS exposure and little or no BAG3 was lost in BAG3^LAAA^ cells. E. Inhibition of caspases resulted in incomplete loss of BAG3. One hour pretreatment with 85 µM zVAD, followed by 2 µM STS up to 20 h, protected BAG3 as did the combination of caspase3 and 10 inhibitors; specific inhibitors concentrations were 40 µM for caspases 3 and 9, and 50 µM for caspase 10. The antibody against cleaved caspase 3 is selective to the cleaved forms and detects two active cleaved species with apparent mobility of 19 and 14 kDa. This latter species is described a further cleavage product. The antibody to caspase 10 detects only the uncleaved, total caspase 10. Arrows indicate target protein bands.

BAGs 3, 4, and 6, all susceptible to STS-mediated degradation, have structural similarities not present in BAG1. Conversely, the STS-resistant BAG1 lacks domains present in BAGs 3, 4 and 6, such as the proline rich region PXXP. We examined whether deletion of the PXXP domain in BAG3 would affect STS-induced loss of BAG3 protein. Stable single cell MDA435 clones overexpressing full length (FL) or deleted (d)PXXP BAG3 [7], [12] were exposed to STS. Progressive degradation of BAG3 was observed beginning as early as 4 hr in FL cells (Figure 3A). In contrast, loss of dPXXP-BAG3 was delayed beyond 12 hr. We obtained similar results in HeLa cells with forced expression of EGFP-dPXXP-BAG3 (Figure 3B). This indicates that the effect is neither cell line nor construct-specific, and suggests that the PXXP domain may contain a target site for BAG3 degradation.

The loss of BAG3 upon activation of the intrinsic apoptosis pathway infers BAG3 may be a target of caspases 3, 9 or 7. *In silico* analysis (ExPASy; http://us.expasy.org/tools/peptidecutter/) did not identify canonical caspase cleavage sites in BAG3. However, two non-canonical putative caspase-cleavage sites were suggested by sequence inspection, ^344^KEVD in the PXXP domain and the C-terminal ^515^LEAD (Figure 3C). KEVD is homologous to the caspase 3 DEVD substrate recognition and cleavage site, and LEAD to the caspase 9 site LEHD [13]. These sites are present in BAG6 (^998^DEQD [14]) and BAG4 (^421^LELD), respectively, but not in BAG1. The BAG3 KEVD/LEAD sites are conserved in upper mammals (Figure 3C, table inset). The importance of these sites is suggested by the conservation of potential caspase 3 (^268^SEVD) and caspase 9 (^508^LKLD) sites in the Drosophila BAG ortholog *EVIL/starvin* (*svn*, CG32130, FBgn00867) [15].

Alanine mutagenesis was used to neutralize the putative caspase sites, and stable bulk clones of BAG3^KAVA^, BAG3^LAAA^, and wild type (B3) expressing MDA435 cells were generated. STS treatment resulted in loss of BAG3 in the B3 cells, whereas loss was limited in BAG3^KAVA^ cells and minimal in BAG3^LAAA^ (Figure 3D). These data argue that ^344^KEVD and ^515^LEAD are functional caspase recognition and cleavage sites in BAG3. We further examined caspase involvement in BAG3 proteolysis using selective and pan-caspase inhibition. The broad caspase inhibitor Z-Val-Ala-Asp(OMe) fluoromethyl ketone (zVAD) abrogated caspase activation and loss of BAG3 in the presence of STS (Figure 3E). Selective inhibition of caspases 3, 10, or both partially prevented BAG3 proteolysis consistent with caspase3 involvement. Notice that the antibody against caspase 3 is selective to the cleaved forms and detects two active cleaved species with apparent mobility of 19 and 14 kDa; the antibody to caspase 10 detects only the uncleaved, total caspase 10, which disappears upon cleavage stimulated by STS treatment. Caspase 10 activation is only partly protected by individual caspase inhibitors and maximally protected by zVAD. Taken together, these results indicate a caspase-dependent cleavage of BAG3 but suggest that additional steps may be needed for complete BAG3 loss.

### BAG3 is ubiquitinated and degraded by the proteasome

**Figure 4:**
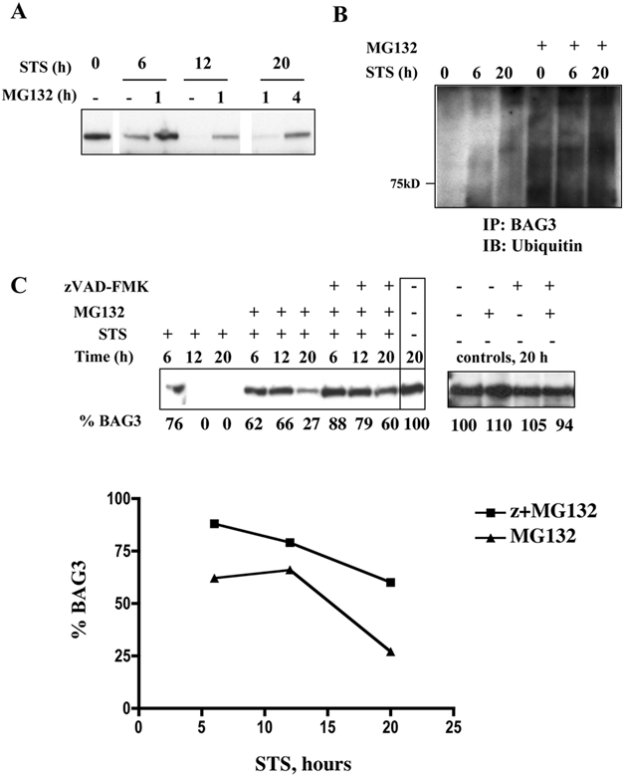
Sequential inhibition of caspases and proteasome provides collaborative protection of BAG3. A. Proteasome inhibition provides dose-dependent partial protection of BAG3. FL-BAG3 cells were pretreated with 20 µM MG-132 for 1 or 4 hrs prior to exposure to 2 µM STS. Inhibition of proteasomal degradation by pretreatment with MG-132 prior to STS exposure reduced BAG3 degradation. B. BAG3 is ubiquitinated in response to STS treatment. FL-BAG3 cells were treated with 2 µM STS alone or following 4 hr preincubation with 20 µM MG-132. Lysates were immunoprecipitated with anti-BAG3 and the immunoblots were probed for ubiquitin. BAG3 was ubiquitinated and degraded in the absence of proteasome inhibition. C. STS-induced loss of BAG3 is minimized by the combination of caspase and proteasomal inhibition. FL-BAG3 cells were preincubated with 0.05% DMSO vehicle or 20 µM MG-132 for 4 hours with or without 1 additional hour of zVAD preincubation (85 µM). Treatments were followed by a total STS exposure of up to 20 hrs. A progressive retention of BAG3 was seen with inclusion of each inhibitor. Relative levels of BAG3, determined using ImageJ™, are indicated below the respective blots and plotted as percent remaining BAG3 with increasing STS time.

Proteasomal degradation is a major protein regulatory mechanism [16], [17]. Chemical inhibitors of the 20S subunit of the proteasome, such as lactacystin and MG-132, prevent degradation of polyubiquitinated proteins [18]. Neither mutation of the KEVD caspase-recognition site, nor pharmacological inhibition of selected caspases afforded complete protection of BAG3 in the face of intrinsic apoptosis pathway activation, suggesting complementary proteolytic mechanisms. Addition of MG-132 to STS resulted in near complete BAG3 retention (Figure 4A). MG-132 pretreatment in the absence of STS caused accumulation of poly-ubiquitinated BAG3, indicating that some BAG3 ubiquitination occurs basally (Figure 4B, lane 1). Augmented ubiquitination of BAG3 was demonstrated with the combination of STS and MG-132 arguing that BAG3 is targeted to the proteasome under STS stress (Figure 4B, lane 4 v. lane 1). We next investigated the complementarity of pharmacological inhibition of both caspases and the proteasome on BAG3 rescue. Some protection of BAG3 was seen with proteasome inhibition alone (Figure 4C), while there was no BAG3 degradation when zVAD and MG-132 were used in combination. A plot of these data demonstrates progressive loss of BAG3 in the presence of MG-132. This is attenuated with the combination of zVAD and MG-132. These results were recapitulated when lactacystin was used as a proteasomal inhibitor. Disappearance of BAG3 signal caused by STS was prevented by pre-treatment with lactacystin (Figure S3A). The combination zVAD and lactacystin prevented loss of BAG3 in FL and dPXXP cells, while minimally affecting the already resistant dPXXP form (Figure S3B). Taken together these data suggest that both caspase cleavage and proteasomal degradation are required for the degradation of BAG3 in STS-driven apoptosis and that the role of caspase cleavage may be primary.

### Noncleavable BAG3 is protected from STS-induced ubiquitination and degradation

**Figure 5:**
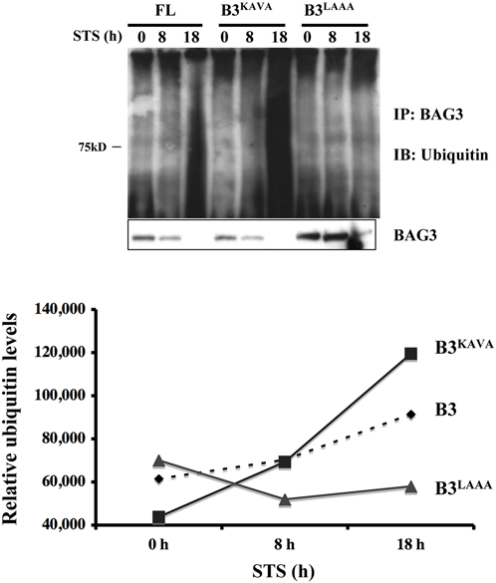
BAG3 degradation requires sequential caspase cleavage followed by ubiquitination. Mutation of the LEAD caspase cleavage site precludes ubiquitination and loss. B3, BAG3^KAVA^ and BAG3^LAAA^ cells were exposed to 2 µM STS as indicated. Iodoacetamide-treated lysates were immunoprecipitated with anti-BAG3 and immunoblots probed for ubiquitin. The partial protection of BAG3^KAVA^ results in a greater quantity of polyubiquitinated BAG3. Minimal ubiquitination of BAG3^LAAA^ is demonstrated, consistent with a requirement for cleavage prior to ubiquitination. Quantity of polyubiquitinated BAG3 protein was estimated by densitometry.

Our data implicate both caspase cleavage and ubiquitination in the regulation of BAG3 quantity. The suggestion that caspase cleavage has a primary role in BAG3 degradation led us to propose that the process is sequential, with one mechanism as a prerequisite for the other. We thus tested whether caspase site mutants, BAG3^KAVA^ and BAG3^LAAA^, are prone to ubiquitination. BAG3^LAAA^ was minimally ubiquitinated. Ubiquitination changed little over the time course of STS exposure, remaining commensurable with that seen during basal turnover of BAG3 (Figure 5). Progressive ubiquitination of BAG3^KAVA^ was observed, maximal at 20 hr, as more of the BAG3 substrate became available due to impaired caspase cleavage. These data demonstrate that caspase cleavage of BAG3 is necessary for stimulated ubiquitination and degradation.

### Noncleavable BAG3 protects cells from STS-induced apoptosis

**Figure 6:**
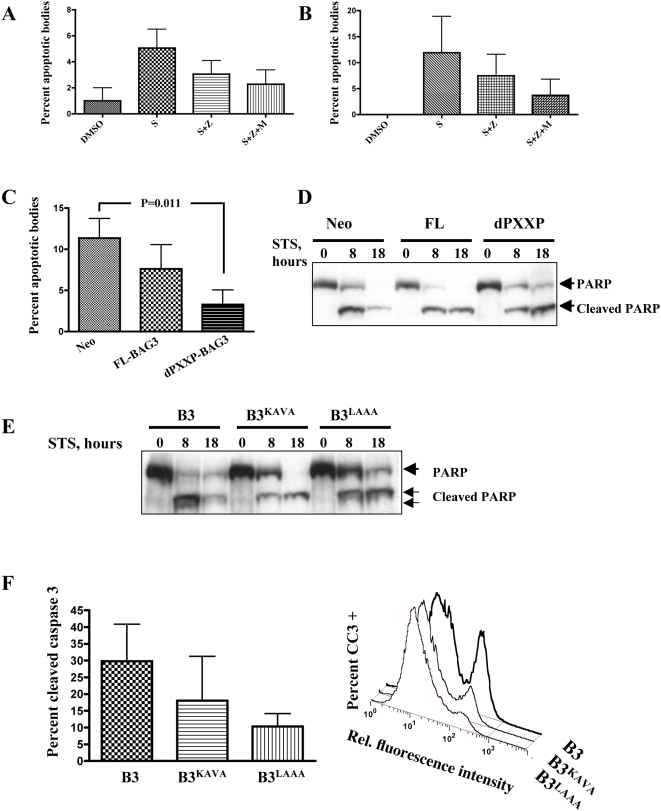
Absence of caspase cleavage of BAG3 confers cellular protection against STS-mediated apoptosis. A, B. STS-induced apoptosis was reduced with caspase and proteasome inhibition. Cells were pretreated with MG-132 for 4 hrs and treated with 2 µM STS and 85 µM zVAD-fmk for an additional 8 hrs. Wild type MDA435 (A) and FL-BAG3 overexpressors were fixed and stained with DAPI and apoptotic bodies scored from 3–5 independent fields; data represent mean and SEM (n = 2). C. Apoptosis is delayed in dPXXP-BAG3 lacking ^344^KEVD. At 8 hours of STS exposure, reduction in apoptotic bodies is seen in FL-BAG3 overexpressing cells and in dPXXP-BAG3 cells; 11–14 random fields were scored, data represent mean and SEM. D. PARP cleavage and loss of total PARP is reduced in dPXXP-BAG3 cells. STS exposure resulted in reduced PARP cleavage in FL cells compared to Neo controls, and both reduced and delayed PARP cleavage in dPXXP-BAG3 cells. E, F. Reduced apoptosis occurs in caspase-resistant BAG3 transfectants. FL-BAG3 wild type, BAG3^KAVA^ and BAG3^LAAA^ were treated with 2 µM STS or 0.05% DMSO vehicle control as shown. Delayed and reduced PARP cleavage is demonstrated in cells overexpressing KEVD and LEAD BAG3 mutants. Cleaved caspase-3, an earlier apoptotic event, was measured by flow cytometry after 6 h STS treatment. As with PARP cleavage, BAG3^LAAA^ had the least cleavage of caspase 3, the right panel shows a representative histogram; data represent mean and SEM (n = 2).

Our data showing silencing of BAG3 increased susceptibility to STS-mediated apoptosis led us to ask if the caspase-resistant mutants conferred cytoprotection. Apoptotic bodies in DAPI-stained STS-treated cells were counted in wild type MDA435 and MDA_FL overexpressors. A progressive reduction in apoptotic bodies was observed upon STS treatment with added zVAD, or zVAD plus MG-132, supporting a role for the loss of BAG3 augmenting STS cell injury (Figure 6A,B). Similarly, dPXXP cells lacking the KEVD sequence underwent significantly less apoptosis than wild type cells or FL-BAG3 (Figure 6C). This decrease in apoptosis is also demonstrated by delayed and reduced PARP cleavage and total PARP loss under the same conditions (Figure 6D). PARP cleavage was reduced and delayed in BAG3^KAVA^ and total PARP was only minimally reduced in BAG3^LAAA^ cells, consistent with cellular protection. The intermediate resistance to STS apoptosis of BAG3^KAVA^ is also demonstrated by reduced caspase 3 activation, which decreased progressively from B3 to BAG3^KAVA^ to BAG3^LAAA^ cells (Figure 6F). Collectively these results indicate dose-dependent cytoprotection from apoptosis is afforded by BAG3. When over expressed BAG3 delays, but does not abrogate, cellular commitment to apoptosis. Prevention of BAG3 degradation through blockade of caspase cleavage and subsequent ubiquitination results in cellular protection from STS-mediated apoptosis.

## Discussion

Survival is controlled by the balance of information within the cell in response to gene expression, protein translation, and protein and message stability as a function of the cellular microenvironment. Cellular and environmental stresses can lead to apoptosis. The two most common mechanisms for protein loss in apoptosis are direct caspase cleavage, and proteasomal degradation of ubiquitinated proteins [3], [19], [20]. The prosurvival protein, BAG3, protects cells from apoptosis caused by heat [5], [7]. We hypothesized that decreased cellular survival in response to STS was due to destruction of BAG3 protein through STS-stimulated caspase activation. This was confirmed through demonstration that BAG3 was lost in response to STS, in a time- and dose-dependent fashion, temporally correlated with activation of caspases 3, 9, and 7. The involvement of caspases in BAG3 degradation was established by protection of BAG3 in the presence of the pan-caspase inhibitor zVAD and by mutagenesis of putative caspase recognition sites. However, BAG3 was not completely protected by individual caspase inhibitors or by mutation of the caspase 3 site KEVD (BAG3^KAVA^). This led to the conclusion that other proteolytic events were required. Inclusion of proteasome inhibitors provided partial protection of BAG3 protein that was complementary with zVAD. BAG3 was polyubiquitinated in MDA435 cells in the absence of stress, consistent with proteasome involvement in basal BAG3 turnover; progressive ubiquitination and subsequent BAG3 loss was observed with STS. Little or no ubiquitination of caspase-resistant BAG3^LAAA^ was observed, while increased ubiquitination was seen in BAG3^KAVA^ as expected because of its partial protection from caspase cleavage and loss. Since absence of BAG3 cleavage precluded ubiquitination, these data argue a requirement for sequential caspase cleavage followed by ubiquitination. Protection of BAG3 translated into cellular protection against STS-induced apoptosis. In forced BAG3 expression experiments, cytoprotection correlated with the molecular construct most resistant to degradation. In contrast, silencing BAG3 augmented STS-mediated apoptosis. Together these data confirm that BAG3 functions as a pro-survival protein, the presence of which is regulated by caspase cleavage followed by ubiqutination and proteasomal degradation. It further suggests that impaired BAG3 degradation is central in the protection of cancer cells against intrinsic apoptotic pathway stress.

Many important signaling proteins are controlled by polyubiquitination, including p53 [21], p21^cip1^ [22], c-Jun [23], and c-fos [24]. These proteins are targeted for polyubiquitination and chaperoned to the proteasome by heat shock proteins [25], [26] for subsequent de-ubiqutination and cleavage [3], [7]. Other proteins, such as APAF-1 [27] or MDM2 [28], are regulated by caspase cleavage. Relatively few proteins, such as the small heat shock co-chaperone p23 [17], are regulated by both caspases and the proteasome. Many other important survival proteins are regulated by either caspases or the proteasome (but not both) in different cellular contexts. Examples among this group are AKT [1], [7], Bcl2 and Bcl_XL_ [2]. However, it remains unclear whether regulation of these proteins requires involvement of both the caspase and proteasome pathways as is shown herein for regulation of BAG3.

The evidence for caspase cleavage of BAG3 is strong, even in the absence of canonical caspase binding and cleavage sites. Two putative caspase recognition and cleavage sites, each containing the ExD sequence [13], [29]–[31], were identified *in silico* and then confirmed functionally in human BAG3. These sites were relatively well conserved in mammalian BAG3 and in BAGs 4 and 6, the latter having been reported to be functional [14]. The caspase 3-like motif K/RexD and the caspase 9-like LexD are conserved in BAG3 in higher mammals. The caspase 3 motif is also present in the Drosophila Evil/Starvin [15] as SEVD, although the caspase 9-like peptide LKLD, more important to hBAG3 stability per our studies, may not be functional.

Drosophila Starvin was initially identified by Coulson and colleagues in a search for binding proteins to Polycomblike protein [15] but when further analyzed was found to have similarity to human BAGs 3, 4 and 5. We had reported the same Drosophila protein, identified as Evil (Frueauf et al. (2005) Cloning of evil: Characterization of Drosophila melanogaster BAG-family homologues. AACR Meeting Abstracts 2005: 825-a), as the Drosophila BAG3/4 homolog and demonstrated conservation of Hsp70 binding. Further, we have shown that when expressed ectopically in human cancer cells, Evil/Starvin is also a stress response survival protein (Frueauf, Virador, Kohn, manuscript in preparation). Starvin functions as a stress response and survival protein in flies with a phenotype that has similarity to two different BAG3 knockout mice [32], [33]. These findings confirm the conserved importance of BAG3/Evil/Starvin protection for selected cell survival.

We and others showed that BAG3 overexpression provided cytoprotection from a variety of stresses. Overexpression of BAG3, but not BAG domain deletion mutants, protected from heat shock [7]. BAG3 overexpression, alone or in combination with ectopic expression of bcl2, overcame bax-mediated apoptosis [5]. The bax/bcl2 apoptotic machinery is linked to the intrinsic apoptotic pathway and the overexpression of BAG3 in those experiments may have been sufficient to overcome that degree of caspase activation. This is similar to our findings that overexpression of BAG3 yields some reduction in apoptotic bodies in response to STS, even more prominently in the practically protected dPXXP BAG3 (Figure 6). Loss of BAG3 by silencing also augmented cell death in response to STS. This same increased apoptosis was reported when BAG3 was silenced in acute and chronic lymphocytic leukemia cells [34], [35]. Thus, protection of BAG3 from proteolysis has a selective anti-apoptotic outcome, interacting with chaperone and non-chaperone pro-survival pathways in stressed cancer cells.

## Materials and Methods

### Cell culture and transfectants

The MDA435 human breast cancer cell line and stable transfectant single cell clones containing full length BAG3 (FL) and PXXP domain-deleted BAG3 (d)PXXP-BAG3 have been reported [7], [12]. These constructs were subcloned into pEGFP-C1 (BD Biosciences, Palo Alto, CA) for transfection into HeLa cells. EGFP transfectants were sorted by FACS to enrich for GFP-positive cells. Site-directed mutagenesis was used to generate BAG3^E345A/D347A^ (BAG3^KAVA^) and BAG3^E516A/D518A^ (BAG3^LAAA^; Bio S&T Inc, Montreal, CAN). Unless otherwise indicated all transfectants were selected in bulk. Cells were cultured in maintenance concentrations of G418 until one passage prior to experiments. All experiments were performed in triplicate.

### Cell lysates, immunoblotting and immunoprecipitation

All experiments were done with subconfluent cells. STS or 0.05% DMSO vehicle control were added for the times and concentrations noted. Floating and attached cells were pooled for all analyses. Cell lysis and immunoblotting were performed as reported [7]. Iodoacetamide 10 mM was added to the modified RIPA lysis buffer for studies of ubiquitination [7]. Urea 4 M was added to the sample buffer for PARP immunoanalysis and samples were heated at 95°C for 7 min prior to loading. Anti-ubiquitin (Ub) and -PARP antibodies were from StressGen (Victoria BC, Canada); antibodies against caspase 10, cleaved (cl) caspase-3 (Asp175), cl caspase-7 (Asp198), cl caspase-8 (Asp384), and cl caspase-9 (Asp330) were from Cell Signaling (Beverly, MA). Antibodies to GAPDH and β-tubulin were from Abcam (Cambridge, MA). (Antibodies to BAG1 and BAG4 were from Santa Cruz Biotechnology, Inc (Santa Cruz, CA); anti-peptide BAG3 and BAG6 antibodies have been reported [7], [36]. Specific caspase inhibitors were from Calbiochem (San Diego, CA), all other chemicals from Sigma (St. Louis, MO).

### Immunofluorescence microscopy

Cells were plated on uncoated glass coverslips and allowed to secrete and adhere to their own extracellular matrix for 2–3 days. After treatment, cells were fixed in 4% formaldehyde, rinsed in PBS and blocked for 1 hr in PBS containing 3% BSA. Cells were stained with antibodies to BAG3 (1∶500 dilution) and counterstained with DAPI (Vector laboratories, Burlingame, CA). Where indicated, cells were loaded with MitoTracker Red CMXRos (Invitrogen, CA), following manufacturer's recommendations. Fluorescence was examined with a Zeiss LSM 510 Confocal Microscope (Carl Zeiss Inc, Thornwood, NY, USA) using a 40× 1.3 NA Plan Neofluar objective. To assess apoptosis the number of cells containing apoptotic bodies was expressed as a percent of the total cells visualized.

### Flow cytometry

Intracellular protein expression was assessed according to manufacturer's protocol (Cell Signaling, Inc). Permeabilized cells were incubated with antibodies against cl caspase-3 1∶200 or BAG3 (1∶500 dilution) followed by Alexa 488-conjugated secondary (1∶200 dilution). The same protocol was used for cl-PARP with a FITC mouse anti cl PARP (Asp214) added according to manufacturers instructions (BD-Pharmingen, San Jose, CA). Analysis was carried out on a FACS Calibur flow cytometer (BD Biosciences, San Jose, CA). Data were analyzed using FlowJo (TreeStar Inc, Palo Alto, CA).

### Gene silencing

BAG3 and control (non-silencing) siRNA were purchased from Qiagen, Inc. (Valencia, CA). siBAG3 40 nM or molar equivalent non-silencing siRNA were introduced to cells in siLentFectTM lipid reagent (Bio-Rad, Hercules, CA) as described [12]. Reagents were added to the cells after up to 96-hour siRNA incubation and maintained until collection.

### Statistical analysis

Numerical values are expressed as mean±SEM of replicate experiments unless otherwise indicated. Comparisons between two groups were made by unpaired Student's *t*-test using GraphPad Prism software (San Diego, CA). Two-sided p values are reported.

## Supporting Information

Figure S1 A fraction of BAG3 co-localizes with mitochondria in early STS-mediated stress. BAG3 stained cells (green) loaded with Mito Tracker (red) and counterstained with DAPI (blue). Arrows show colocalization in yellow. At higher STS doses, there is loss of the BAG3 green signal and a generalized uptake of Mito Tracker in cell nuclei (arrow head) indicating loss of both nuclear and mitochondrial membrane integrity.
(5.22 MB TIF)

Figure S2 BAG3 is transiently induced in early STS-mediated stress. A. Flow cytometry of MDA435 cells stained for BAG3 (secondary Alexa 666 anti Rabbit) and subject to increasing STS doses. B. MDA435 cells stained for BAG3 after 4 h STS exposure (secondary Alexa 594 anti Rabbit).
(3.57 MB TIF)

Figure S3 Inhibition of caspases and proteasome provides collaborative protection of BAG3. A. Proteasome inhibition with lactacystyin (1 µM, 4 hr preincubation) in MDA-435 cells overexpressing BAG3 or BAG3-dPXXP provides partial protection of BAG3. B. Lactacystin in combination with zVAD provides near full protection of BAG3, similar to MG-132.
(0.29 MB TIF)

Figure S4 Disappearance of the BAG3 signal by siRNA. MDA435-Neo cells were exposed to 200 nM BAG3 siRNA or scramble control for 72 hours followed by addition of vehicle control or 2µM STS for additional 6 hrs. Immunoblot demonstrates silencing of BAG3.
(0.17 MB TIF)

Figure S5 Commonly used ‘housekeeping proteins’ are degraded by STS in HeLa and MDA 435 cells. A. HeLa cells overexpressing BAG3. BAG3 signal disappears with increasing time of exposure to 2 µM STS. GAPDH (re-blot) likewise disappears with STS exposure. B. MDA435 cells. BAG3 signal disappears with STS and is protected by addition of MG-132 or MG-132 and zVAD in combination. The signal for β-tubulin (re-blot) diminishes with STS exposure.
(0.39 MB TIF)
