# Supplementary figures and images for: The Anti-Apoptotic Activity of BAG3 Is Restricted by Caspases and the Proteasome
Source: PLoS One. 2009 Apr 8;4(4):e5136. doi: 10.1371/journal.pone.0005136 (PMC2662420; doi:10.1371/journal.pone.0005136)

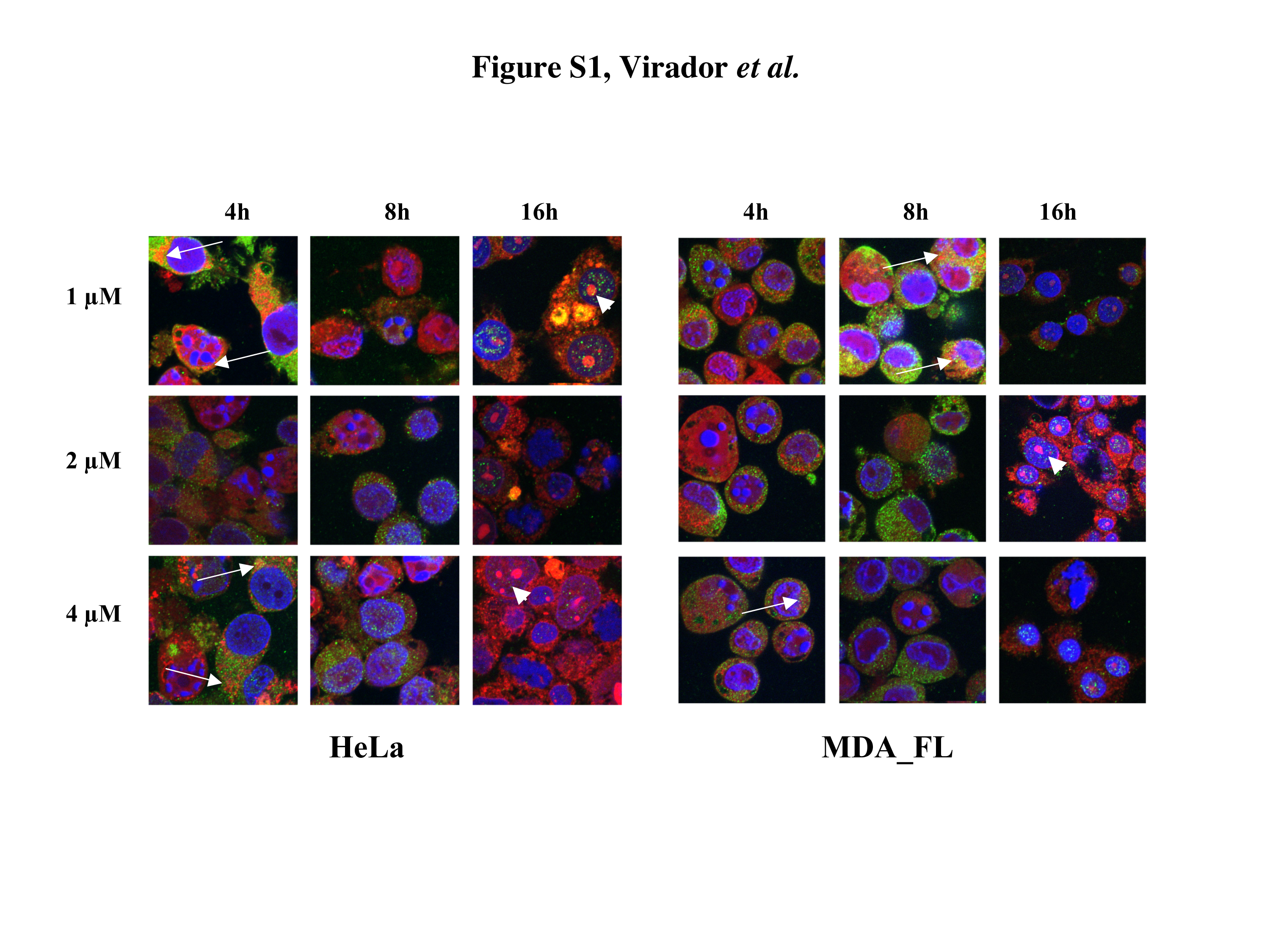

Supplement: Figure S1 — A fraction of BAG3 co-localizes with mitochondria in early STS-mediated stress. BAG3 stained cells (green) loaded with Mito Tracker (red) and counterstained with DAPI (blue). Arrows show colocalization in yellow. At higher STS doses, there is loss of the BAG3 green signal and a generalized uptake of Mito Tracker in cell nuclei (arrow head) indicating loss of both nuclear and mitochondrial membrane integrity. (5.22 MB TIF) [file pone.0005136.s001.tif]

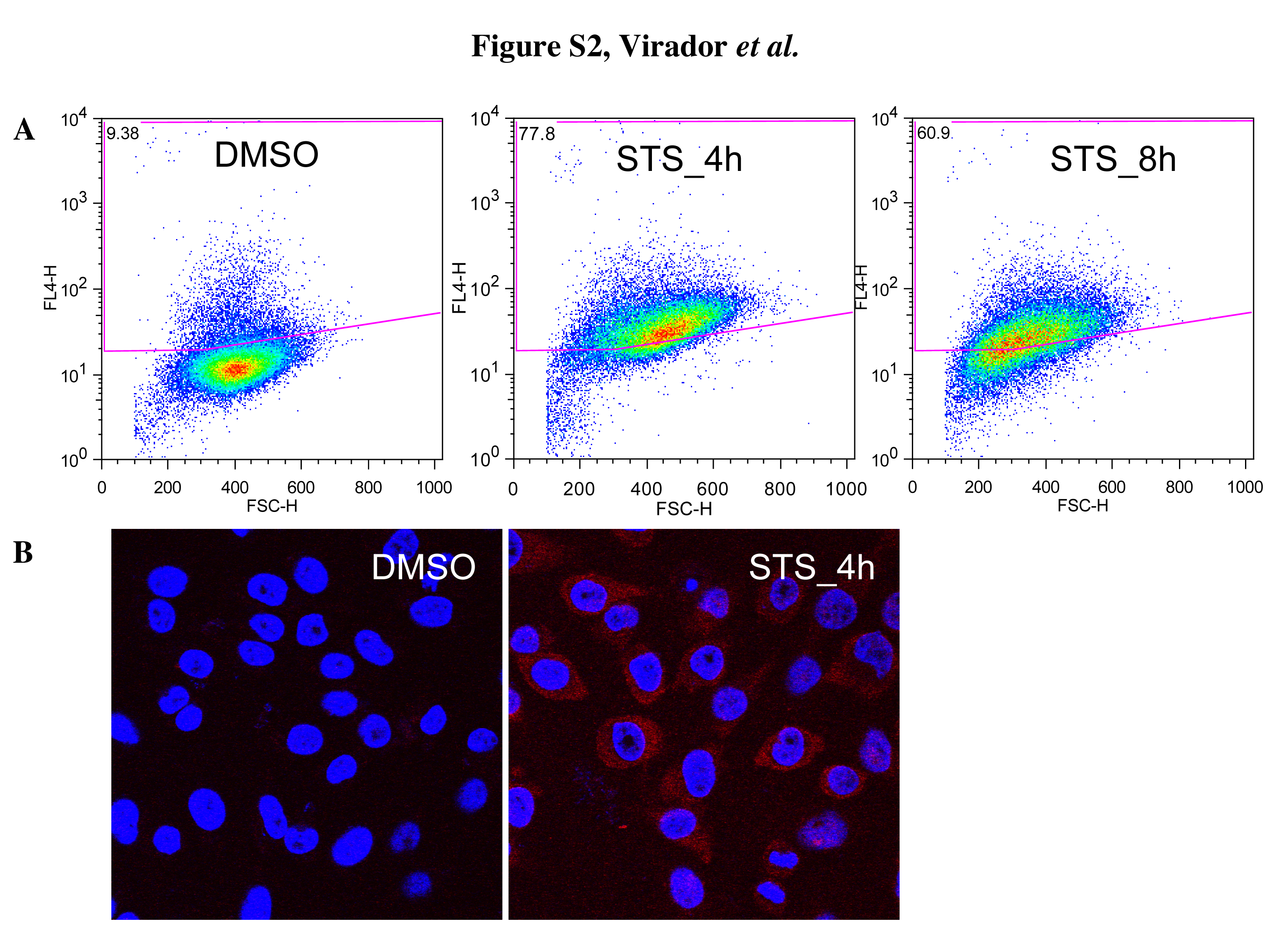

Supplement: Figure S2 — BAG3 is transiently induced in early STS-mediated stress. A. Flow cytometry of MDA435 cells stained for BAG3 (secondary Alexa 666 anti Rabbit) and subject to increasing STS doses. B. MDA435 cells stained for BAG3 after 4 h STS exposure (secondary Alexa 594 anti Rabbit). (3.57 MB TIF) [file pone.0005136.s002.tif]

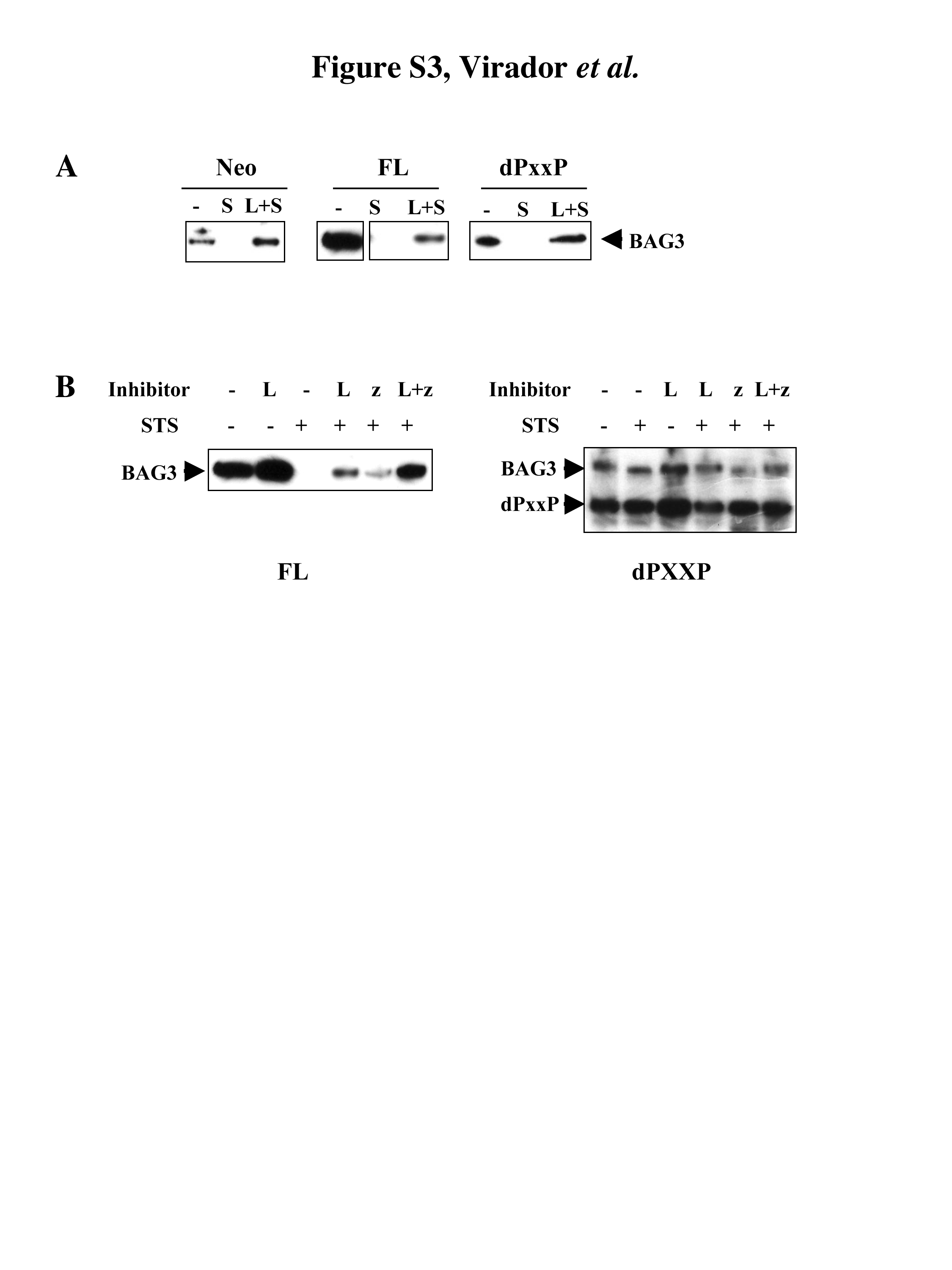

Supplement: Figure S3 — Inhibition of caspases and proteasome provides collaborative protection of BAG3. A. Proteasome inhibition with lactacystyin (1 µM, 4 hr preincubation) in MDA-435 cells overexpressing BAG3 or BAG3-dPXXP provides partial protection of BAG3. B. Lactacystin in combination with zVAD provides near full protection of BAG3, similar to MG-132. (0.29 MB TIF) [file pone.0005136.s003.tif]

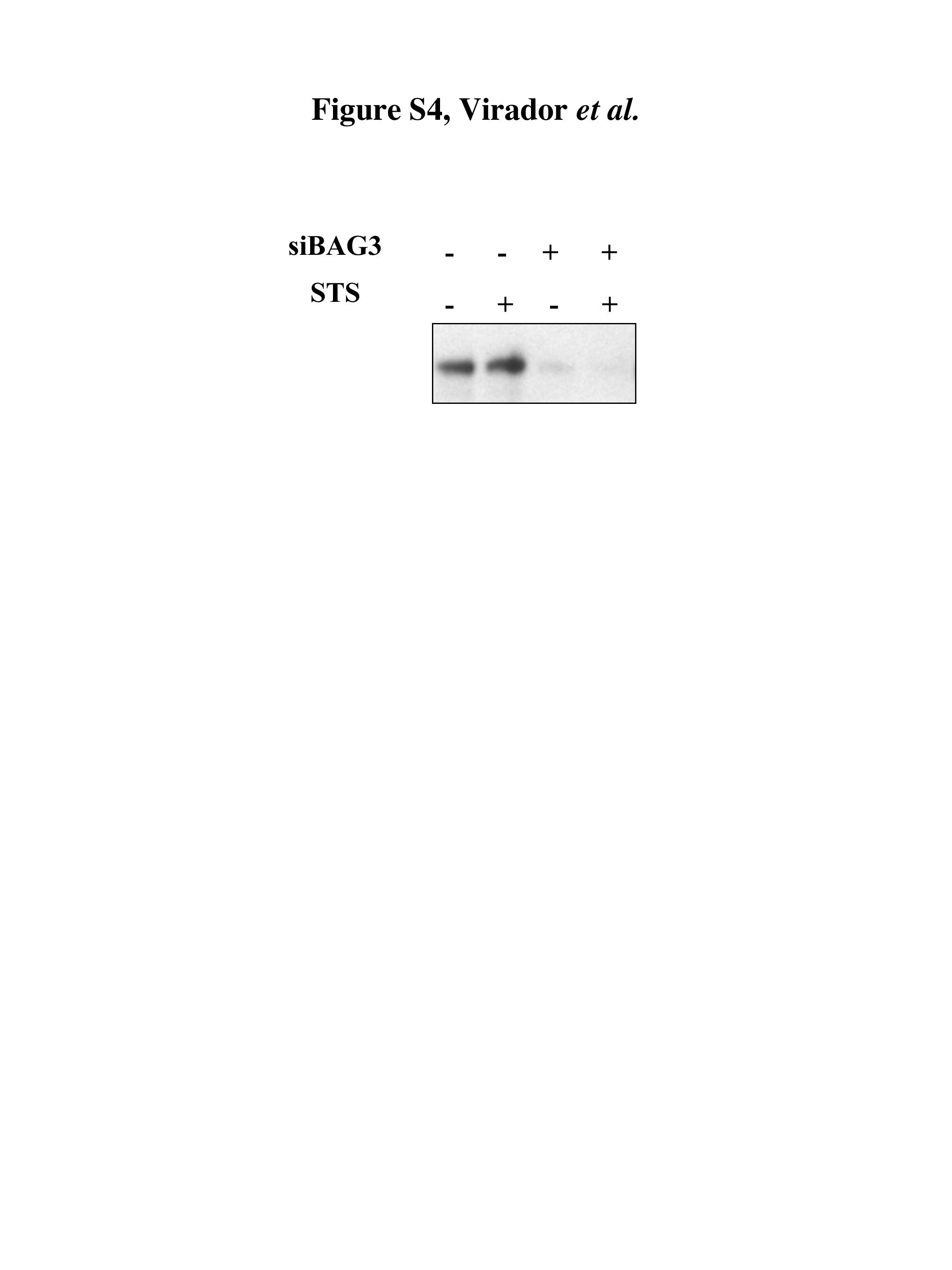

Supplement: Figure S4 — Disappearance of the BAG3 signal by siRNA. MDA435-Neo cells were exposed to 200 nM BAG3 siRNA or scramble control for 72 hours followed by addition of vehicle control or 2µM STS for additional 6 hrs. Immunoblot demonstrates silencing of BAG3. (0.17 MB TIF) [file pone.0005136.s004.tif]

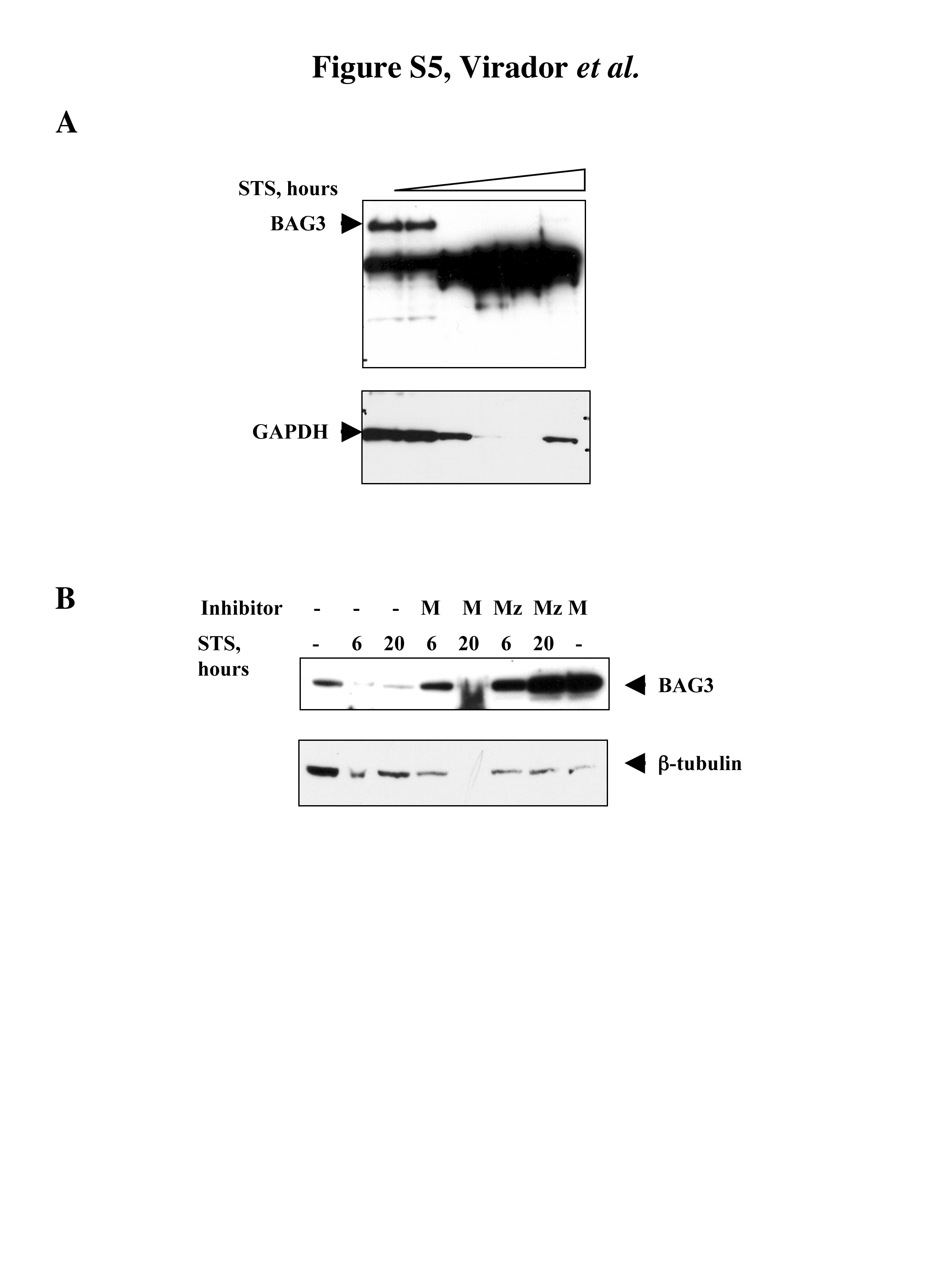

Supplement: Figure S5 — Commonly used ‘housekeeping proteins’ are degraded by STS in HeLa and MDA 435 cells. A. HeLa cells overexpressing BAG3. BAG3 signal disappears with increasing time of exposure to 2 µM STS. GAPDH (re-blot) likewise disappears with STS exposure. B. MDA435 cells. BAG3 signal disappears with STS and is protected by addition of MG-132 or MG-132 and zVAD in combination. The signal for β-tubulin (re-blot) diminishes with STS exposure. (0.39 MB TIF) [file pone.0005136.s005.tif]
